# Artificial intelligence in prostate MRI: Comparative diagnostic performance in a high-prevalence cohort

**DOI:** 10.1177/02841851261470455

**Published:** 2026-07-27

**Authors:** Nádia Gonçalves Ferreira, Owen Matthew Truscott Thomas, Kjell-Inge Gjesdal, Stig Müller, Jan Oldenburg, Ingrid Framås Syversen, Anders Christian Hansen, Jonn Terje Geitung

**Affiliations:** 1 Medical Faculty, 60483 University of Oslo, Oslo, Norway; 2 Department of Radiology, Akershus Univesity Hospital, Lørenskog, Norway; 3 Health Services Research Unit, 60483 Akershus University Hospital, Lørenskog, Norway; 4 Sunnmære MR-Klinikk, Ålesund, Norway; 5 Department of Urology, 60483 Akershus University Hospital, Lørenskog, Norway; 6 Department of Oncology, 60483 Akershus University Hospital, Lørenskog, Norway; 7 Department of Applied Mathematics and Theoretical Physics, 151954 University of Cambridge, Centre for Mathematical Sciences, Cambridge, UK; 8 Institute of Clincal Medicine, 6305 University of Oslo, Oslo, Norway

**Keywords:** Genital/reproductive, magnetic resonance imaging, prostate, adults, computer applications-detection, diagnosis

## Abstract

**Background:**

Artificial intelligence (AI) is increasingly used in prostate cancer diagnostic workflows but remains insufficiently validated in high-prevalence cohorts often encountered in academic referral centers.

**Purpose:**

To compare the diagnostic performance of licensed AI software with routine radiologist readings of prostate MRI, using histopathology as the reference standard.

**Material and Methods:**

In this retrospective study, 1000 patients underwent prostate MRI for suspected prostate cancer (between May 2020 and December 2024), followed by transperineal biopsy; 391 subsequently underwent radical prostatectomy. Diagnostic performance was assessed using sensitivity, specificity, positive predictive value (PPV), negative predictive value (NPV), and accuracy across PI-RADS thresholds. Receiver operating characteristic (ROC) analysis, Cohen's kappa for inter-rater agreement, and paired McNemar's test were performed.

**Results:**

In total, 959 patients (mean age = 69.7 ± 8.4 years) were included; clinically significant prostate cancer (csPCa) was found in 830 (86.5%) patients. AI assigned more cases to PI-RADS 1–2 and fewer to PI-RADS 3 (κ = 0.388; *P* <.001). At the PI-RADS ≥3 threshold, radiologists showed sensitivity 96.0%, specificity 20.9%, accuracy 85.9%, PPV 88.7%, and NPV 45.0%, compared with 91.3%, 37.2%, 84.0%, 90.3%, and 40.0% for AI (*P* <.001). ROC performance was comparable (AUC 0.763 vs. 0.739; *P* = .28). In the prostatectomy subgroup, AI demonstrated a higher false-negative rate (8.4% vs. 4.1%; odds ratio = 3.83, 95% confidence interval [CI] = 1.52–11.51).

**Conclusion:**

AI showed diagnostic performance comparable to that of radiologists for csPCa detection, with no significant difference in AUC. AI reduced indeterminate PI-RADS 3 scores but missed more significant cancers.

## Introduction

Worldwide, prostate cancer (PCa) is the second leading cancer diagnosis in men.^1^ Its incidence continues to rise, and the number of affected individuals is expected to reach approximately 2.9 million by 2040.^2^

Since its first use in 1983, magnetic resonance imaging (MRI) has undergone significant technological and clinical advancements, establishing itself as a crucial tool for diagnosing PCa.^3,4^ Current guidelines recommend performing MRI before biopsy to select patients for further diagnostic confirmation and to minimize the overdiagnosis of indolent disease.^5^ To standardize prostate MRI acquisition, interpretation, and reporting, the Prostate Imaging Reporting and Data System (PI-RADS) was introduced in 2012 and has since been updated to version 2.1.^6,7^ However, despite its widespread adoption, PI-RADS interpretation remains limited by moderate specificity and considerable inter-reader variability.^8–10^

To address these challenges, artificial intelligence (AI) has emerged as a promising adjunct for prostate MRI interpretation. AI-powered tools have shown potential to enhance lesion detection and classification, assist patient stratification, and reduce workload and healthcare costs.^11,12^ However, despite increasing interest, solid clinical evidence supporting their widespread adoption in real-world settings remains limited.^13,14^

The mdprostate software (Mediaire GmbH, Berlin, Germany) is a CE-marked, AI-based tool developed to support automated assessment of prostate MRI.^15^ The software identifies suspicious lesions and assigns PI-RADS v2.1 categories based on biparametric MRI sequences. Although such AI systems have the potential to improve diagnostic consistency and reduce inter-reader variability, the clinical performance of mdprostate compared with radiologist interpretation has not yet been systematically evaluated in routine clinical practice.

Therefore, the aim of the present study was to compare the diagnostic performance of a licensed AI software (mdprostate) with routine radiologist readings of prostate MRI at an academic referral center, using histopathology as the reference standard.

## Material and Methods

### Patients

**Figure 1.**
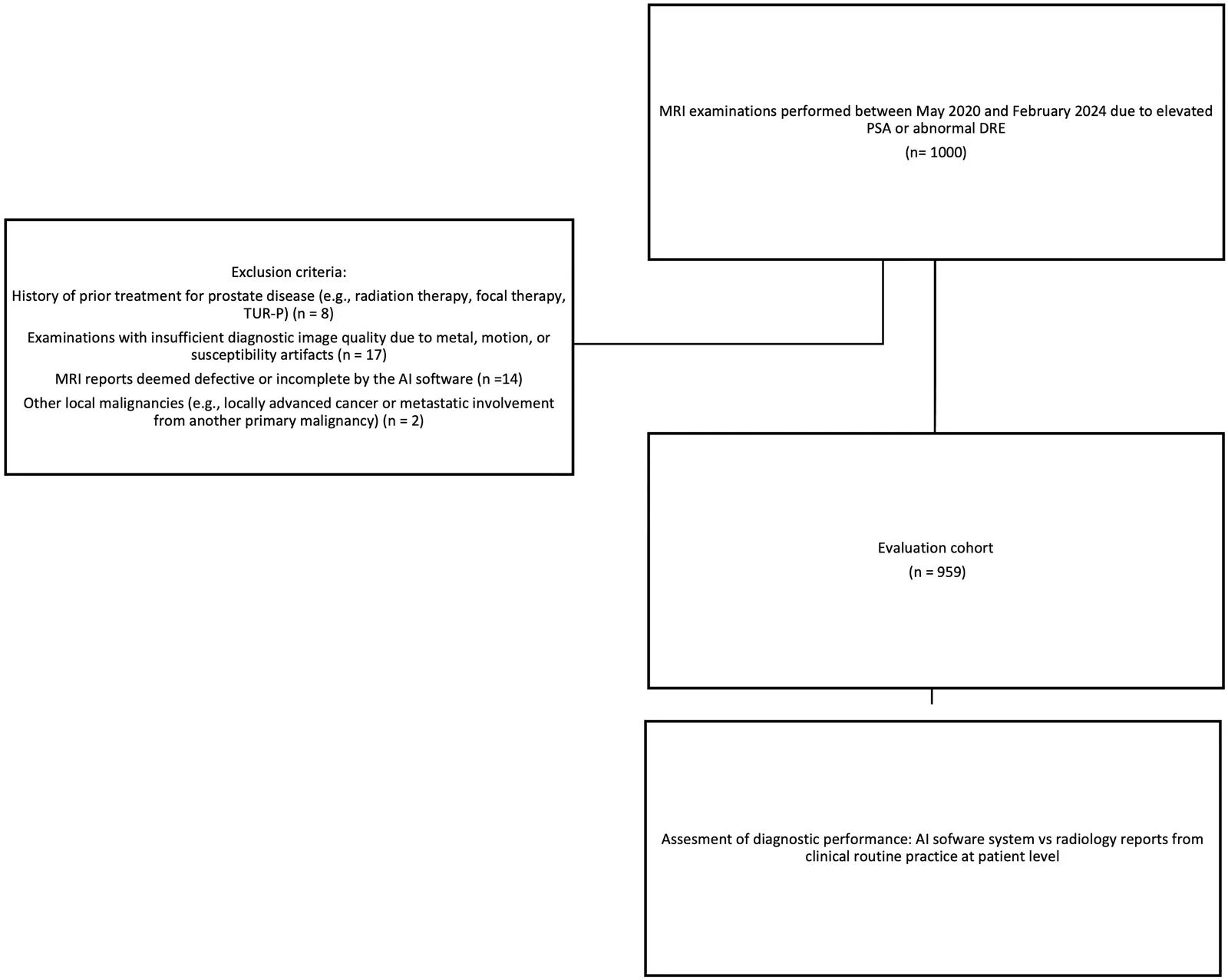
Flow chart of study cohort patients.

*Note.* DRE: digital rectal examination; MRI: magnetic resonance imaging; PSA: prostate specific antigen.

This retrospective single-center study was conducted at our institution. The study was considered outside the scope of the Norwegian Health Research Act by the Regional Ethics Committee (REK; reference no. 627927) and approved by the institutional Data Protection Officer (PVO; 2023_179). Informed consent was not required. All patient imaging was anonymized before AI analysis and handled in accordance with institutional data protection regulations. We retrospectively included 1000 consecutive patients referred for suspected PCa based on abnormal digital rectal examination (DRE) and/or elevated prostate-specific antigen (PSA) levels who underwent both prostate MRI and subsequent transperineal prostate biopsy (TPPB) between May 2020 and February 2024. Histopathologic findings from TPPB served as the reference standard. Of these, 391 patients subsequently underwent radical prostatectomy. No formal sample size calculation was performed; the cohort size was chosen pragmatically to provide a large real-world dataset for evaluating AI-assisted prostate MRI interpretation. Examinations were excluded for prior PCa treatment; inadequate MRI quality independently assessed by two radiologists (with 6 and 30 years of experience) using the prostate imaging quality (PI-QUAL) score^16^; incomplete reports or missing histopathologic data; evidence of locally advanced or metastatic disease from another primary malignancy; or invalid AI-generated reports. After exclusions, 959 patients comprised the final study cohort (Figure 1).

### Imaging

Imaging was obtained using several 3-T (n = 2) and 1.5-T (n = 5) MRI scanners (Philips Medical Systems, the Netherlands), equipped with an anterior coil, with the patient in the supine position. Detailed protocols are described in Supplementary Table S1. A 1.5-T scanner was primarily reserved for patients with metallic implants. Preparation involved a 4-h fast and the self-administration of a rectal micro-enema. In addition, an antispasmodic agent, Butylscopolamine (Buscopan), was given intravenously before the scan to minimize bowel motion. The imaging protocol adhered to the PI-RADS acquisition guidelines, including tri-planar (axial, coronal, and sagittal) T2-weighted (T2W) imaging and axial restricted-field-of-view diffusion-weighted imaging (DWI) with two b-values, including a high b-value of 1500 s/mm^2^. Dynamic contrast-enhanced (DCE) images were acquired simultaneously in 124/959 patients considered high risk due to an initial PSA of ≥20 ng/mL or suspicion of a T-stage tumor ≥2c on DRE. However, not all patients who met the high-risk criteria underwent DCE imaging during their initial assessment (*n* = 36), because the initial protocol assignment in routine clinical practice did not include a dedicated staging protocol with contrast administration. These patients were retained in the analysis because, although DCE may contribute to PCa detection, its formal role within PI-RADS is primarily limited to equivocal peripheral-zone lesions.^17^ MRI examinations were reviewed in routine clinical practice by abdominal radiologists at our institution with 3–20 years of experience in prostate MRI, in accordance with the PI-RADS v2.1 guidelines, using all available MRI sequences, including DCE imaging when acquired.

### AI software

A licensed computer-aided detection (CAD) software (mdprostate software, version 1.3.0; Mediaire GmbH, Berlin, Germany), CE-marked as a Class IIb medical device, was installed on a standalone Picture Archiving and Communication System (PACS) workstation for evaluation.^15^ MRI images were anonymized before analysis. The mdprostate algorithm was developed using biparametric MRI data from 1985 prostate MRI examinations acquired on both 1.5-T and 3-T scanners across multiple centers and vendors. The training dataset included 2044 PI-RADS 4–5 lesions and 404 PI-RADS 3 lesions, with lesion annotations generated using a proprietary automated annotation framework under expert radiologist oversight.^15^ The AI software provided automated zonal segmentation, prostate volume calculation, and lesion detection and classification according to PI-RADS version 2.1, using T2W and DWI. Lesion dimensions and apparent diffusion coefficient (ADC) values were automatically reported but were not included in the analysis. The software generated a standardized two-page report with quantitative data and visual outputs, including MRI images with highlighted lesions and a prostate sector map showing lesion location (Supplementary Figure S2).

### Pathological assessment

All patients underwent TPPB with targeted and systematic sampling at our institution under local anesthesia, performed by experienced urologists using the KOELIS fusion system. Before the biopsy appointment, positive lesions were manually annotated by a radiologist with dedicated expertise in prostate MRI. Patients with negative MRI examinations and clinical suspicion of PCa (abnormal DRE and/or PSA density >0.15) underwent systematic sampling only, in accordance with institutional clinical practice. No additional diagnostic interventions were performed for study purposes. Biopsies were schematically documented on a prostate map to facilitate correlation with pathological findings. Biopsies were interpreted at our institution by experienced consultant uropathologists and visually annotated on a prostate scheme to support correlation between imaging, biopsy location, and histopathology.

### Image and histopathology analysis

Correlation between routine radiology reports, AI-generated MRI reports, and histopathologic findings was performed by consensus of two radiologists with 6 and 30 years of experience in prostate MRI, respectively. Patient-level analyses were conducted using the highest PI-RADS score assigned by the radiologists and the AI software with the corresponding histopathologic biopsy results. Clinically significant PCa (csPCa) was defined as a Gleason score ≥7/International Society of Urological Pathology (ISUP) grade ≥2.^5,18^

### Statistical analysis

We performed a statistical analysis to compare the ability of radiologists’ PI-RADS ratings and the AI software's PI-RADS ratings to predict binarized ISUP ratings, which served as the reference standard for the presence or absence of cancer. The ISUP grade was categorized into <2 and ≥2 to denote the boundary of clinically significant cancer. Different PI-RADS levels were used as thresholds to predict the binary ISUP outcome for both the radiologist and the AI software. Given the binary predictors and outcome, we systematically investigated the true-/false-positive/negative rates by deriving sensitivity, specificity, positive predictive value (PPV), negative predictive value (NPV), and accuracy for each rating system at each threshold. We also reported the results of the McNemar test, designed for 2 × 2 tables with matched subjects, to assess the marginal homogeneity of the radiologist and AI,^19^ providing an odds ratio (OR) with an associated confidence interval for two paired, correlated proportions. In addition, we provided Cohen's κ (kappa) statistic for measuring inter-rater reliability (or degree of consistency) between the raters.^20^ We further presented receiver operating characteristic (ROC) curves to visualize the sensitivity and specificity of the radiologist and AI software, varying with the PI-RADS threshold. We performed a statistical test comparing the area under the curve (AUC) for the radiologist and the AI software, indicating which rater had the best overall predictive performance. For the subgroup analysis according to MRI magnetic field strength (1.5 T vs. 3 T), the AUCs of the AI software were compared using DeLong's test for two independent ROC curves. To assess the precision of the diagnostic accuracy estimates, we conducted a post hoc precision analysis by reporting the full 95% confidence intervals (CIs) for all diagnostic metrics (sensitivity, specificity, PPV, NPV, accuracy, and AUC). Because several CIs were asymmetric on the probability scale, we did not summarize them using a single “margin of error” value; instead, we focused on the total CI width as a direct indicator of precision. The analysis was performed using R version 4.5.2.^21^

## Results

### Patients

The study included 959 patients (mean age = 69.7 ± 8.4 years). Of the 959 MRI examinations, 176 (18.4%) were performed on 1.5-T scanners and 783 (81.6%) on 3-T scanners. Of these, 129 (13.5%) patients showed no histopathological evidence of csPCa, while 830 (86.5%) patients were confirmed to have csPCa, yielding a high-prevalence cohort, with a mean of 1.2 ± 0.6 detected lesions per patient. The median PSA was 8.5 ng/mL (interquartile range [IQR] = 6.6). Detailed information on the distribution of ISUP grades within the study cohort is shown in Table 1. In addition, the distribution of ISUP grades according to the PI-RADS scores assigned by radiologists and the AI software is shown in Table 2.

**Table 1.** Distribution of histopathological grade in the study cohort.

Gleason score	ISUP	*n* (%)
No Pca	No Pca	17 (1.8)
6 (3 + 3)	1	112 (11.7)
7 (3 + 4)	2	334 (34.8)
7 (4 + 3)	3	177 (18.5)
8 (4 + 4 or 3 + 5 or 5 + 3)	4	121 (12.6)
9–10 (4 + 5 or 5 + 4 or 5 + 5)	5	198 (20.6)
		*n* = 959

*Note.* Distribution of histopathological grade among the 959 patients, with the number of individuals (n) and corresponding percentage (%) provided for each ISUP/Gleason score. ISUP: International Society of Urological Pathology; PCa: prostate cancer.

**Table 2.** Patient characteristics and distribution of ISUP grades according to PI-RADS scores.

	No cancer	ISUP 1	ISUP 2	ISUP 3	ISUP 4	ISUP 5
Age, y	52.18 ± 1.12	52.17 ± 1.05	52.18 ± 1.08	52.19 ± 1.02	52.13 ± 1.09	52.17 ± 1.02
PSA	7.48 ± 3.92	7.16 ± 3.61	8.59 ± 4.53	20.22 ± 74.50	15.68 ± 25.20	64.69 ± 197.13
Median	7.10 (IQR 2.40)	6.45 (IQR 3.35)	7.60 (IQR 4.00)	8.70 (IQR 7.50)	9.80 (IQR 6.40)	14.00 (IQR 35.38)
*Radiology reports*						
PI-RADS 1–2	8/60 (13.3)	19/60 (31.7)	26/60 (**43.3**)	1/60 (**1.7**)	4/60 (**6.7**)	2/60 (**3.3**)
PI-RADS 3	**4**/125 (3.2)	**36**/125 (28.8)	60/125 (48)	12/125 (9.6)	7/125 (5.6)	6/125 (4.8)
PI-RADS 4	**5**/232 (2.2)	**33**/232 (14.2)	98/232 (42.2)	50/232 (21.6)	27/232 (11.6)	19/232 (8.2)
PI-RADS 5	0/542 (0.0)	**24**/542 (4.4)	150/542 (27.7)	114/542 (21.0)	83/542 (15.3)	171/542 (31.5)
*AI software*						
PI-RADS 1–2	9/120 (7.5)	39/120 (32.5)	48/120 (**40.0**)	8/120 (**6.7**)	5/120 (**4.1**)	11/120 (**9.2**)
PI-RADS 3	**1**/34 (3.0)	**6**/34 (17.6)	15/34 (44.1)	8/34 (23.5)	3/34 (8.8)	1/34 (3.0)
PI-RADS 4	**3**/165 (1.8)	**34**/165 (20.6)	67/165 (40.6)	32/165 (19.4)	15/165 (9.1)	14/165 (8.5)
PI-RADS 5	**4**/640 (0.5)	**33**/640 (5.2)	204/640 (31.9)	129/640 (20.2)	98/640 (15.3)	172/640 (26.9)
	***n* = 17**	***n* = 112**	***n* = 334**	***n* = 177**	***n* = 121**	***n* = 198**

*Note.* This table presents patient characteristics (age and PSA level) and the distribution of ISUP grades stratified by PI-RADS scores assigned by radiologists in routine clinical practice and by the AI software (*n* = 959). Values are shown as *n* (%) or mean ± SD unless otherwise indicated. False-negatives are underlined and the corresponding percentage are shown in bold, and false-positives are shown in bold. IQR: interquartile range; ISUP: International Society of Urological Pathology; PCa: prostate cancer; PI-RADS: Prostate Imaging Reporting and Data System; PSA: prostate-specific antigen.

### Distribution of PI-RADS scores and inter-rater agreement

**Figure 2.**
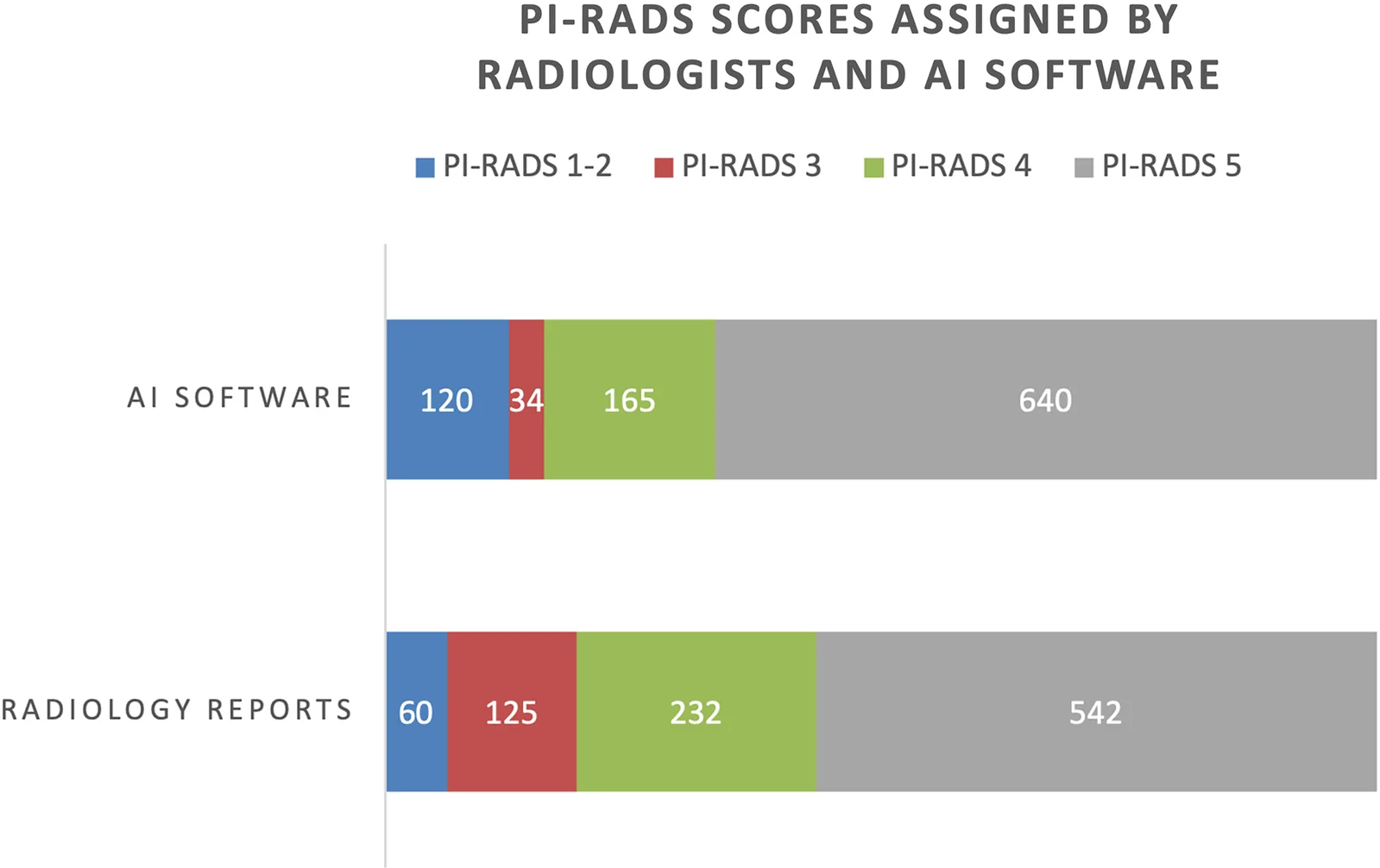
Distribution of the PI-RADS scores assigned by the radiologists and the AI software. The horizontal bar chart shows the distribution of PI-RADS scores assigned by radiologists during routine practice and with the AI software tool (*n* = 959). PI-RADS 1 and 2 are shown in blue, PI-RADS 3 in red, PI-RADS 4 in green, and PI-RADS 5 in gray.

The distribution of PI-RADS scores assigned by radiologists and the AI software is shown in Figure 2. The AI classified a higher proportion of lesions as PI-RADS 1–2 than radiologists (120 [12.5%] vs. 60 [6.3%]) and fewer as indeterminate PI-RADS 3 (34 [3.5%] vs. 125 [13.0%]). Agreement between radiologists and AI for PI-RADS assignment was assessed using Cohen's kappa (κ = 0.388; *P* < .001), indicating fair agreement.

### Diagnostic performance: radiology reports from routine practice vs AI software

Detailed performance metrics—including sensitivity, specificity, PPV, NPV, and diagnostic accuracy—of radiology reports and AI software across PI-RADS thresholds ≥3, ≥4, and 5 at the patient level are summarized in Table 3.

**Table 3.** Diagnostic performance metrics for radiologists and AI software for detecting lesions with csPCa (ISUP ≥2) across the PI-RADS category threshold ≥3, ≥4, and 5.

PI-RADS threshold		**≥3**	**≥4**	**5**
Radiology reports				
ISUP ≥2	Sensitivity	0.960 (0.945–0.972)	0.858 (0.832–0.881)	0.624 (0.590–0.657)
	Specificity	0.209 (0.143–0.290)	0.519 (0.430–0.608)	0.814 (0.736–0.877)
	Accuracy	0.859 (0.836–0.881)	0.812 (0.786–0.837)	0.650 (0.618–0.680)
	PPV	0.887 (0.864–0.907)	0.920 (0.898–0.938)	0.956 (0.935–0.971)
	NPV	0.450 (0.321–0.584)	0.362 (0.293–0.436)	0.252 (0.211–0.296)
AI software				
ISUP ≥2	Sensitivity	0.913 (0.892–0.932)	0.881 (0.857–0.902)	0.727 (0.695–0.757)
	Specificity	0.372 (0.289–0.462)	0.426 (0.340–0.516)	0.713 (0.627–0.789)
	Accuracy	0.840 (0.816–0.863)	0.820 (0.794–0.843)	0.725 (0.695–0.753)
	PPV	0.903 (0.881–0.923)	0.908 (0.886–0.927)	0.942 (0.921–0.959)
	NPV	0.400 (0.312–0.493)	0.357 (0.282–0.438)	0.288 (0.239–0.341)
McNemar *P* value		<.001	.02	<.001

*Note.* This table summarizes the diagnostic performance of radiology reports and AI software in detecting prostate lesions with ISUP grade ≥2 across PI-RADS thresholds of ≥3, ≥4, and 5. The metrics presented include sensitivity, specificity, accuracy, PPV, and NPV. Each value includes a point estimate, with the 95% CI shown in parentheses. McNemar *P* values represent paired comparisons of radiologists vs. AI for binary classification at each PI-RADS threshold. CI: confidence interval; ISUP: International Society of Urological Pathology; NPV: negative predictive value; PI-RADS: Prostate Imaging Reporting and Data System; PPV: positive predictive value.

At a PI-RADS threshold ≥3, radiologists achieved a sensitivity of 96.0% (range = 94.5–97.2) and specificity of 20.9% (range = 14.3–29.0), whereas AI achieved a sensitivity of 91.3% (range = 89.2–93.2) and specificity of 37.2% (range = 28.9–46.2). Overall accuracy was 85.9% (range = 83.6–88.1) for radiologists and 84.0% (range = 81.6–86.3) for AI. AI resulted in approximately 5% more false-negative cases than radiologists (72 vs. 33), but fewer false-positive cases (81 vs. 102), and detected fewer indolent cancers (ISUP grade 1) (73 vs. 93). PPV was similar between radiologists and AI (88.7% [range = 86.4–90.7] vs 90.3% [range = 88.1–92.3]), whereas NPV remained modest (45.0% [range = 32.1–58.4] vs 40.0% [range = 31.2–49.3]).

At a PI-RADS threshold ≥4, radiologists achieved a sensitivity of 85.8% (range = 83.2–88.1) and a specificity of 51.9% (range = 43.0–60.8), while AI achieved a sensitivity of 88.1% (range = 85.7–90.2) and a specificity of 42.6% (range = 34.0–51.6). PPV values were 92.0% (range = 89.8–93.8) for radiologists and 90.8% (range = 88.6–92.7) for AI, with NPV values of 36.2% (range = 29.3–43.6) and 35.7% (range = 28.2–43.8), respectively. Overall accuracy was 81.2% (range = 78.6–83.7) for radiologists and 82.0% (range = 79.4–84.3) for AI.

At a PI-RADS threshold ≥5, AI demonstrated higher sensitivity (72.7% [range = 69.5–75.7] vs. 62.4% [range = 59.0–65.7]) and higher overall accuracy (72.5% [range = 69.5–75.3] vs. 65.0% [range = 61.8–68.0]) compared with radiologists, whereas radiologists showed higher specificity (81.4% [range = 73.6–87.7] vs. 71.3% [range = 62.7–78.9]).

**Figure 3.**
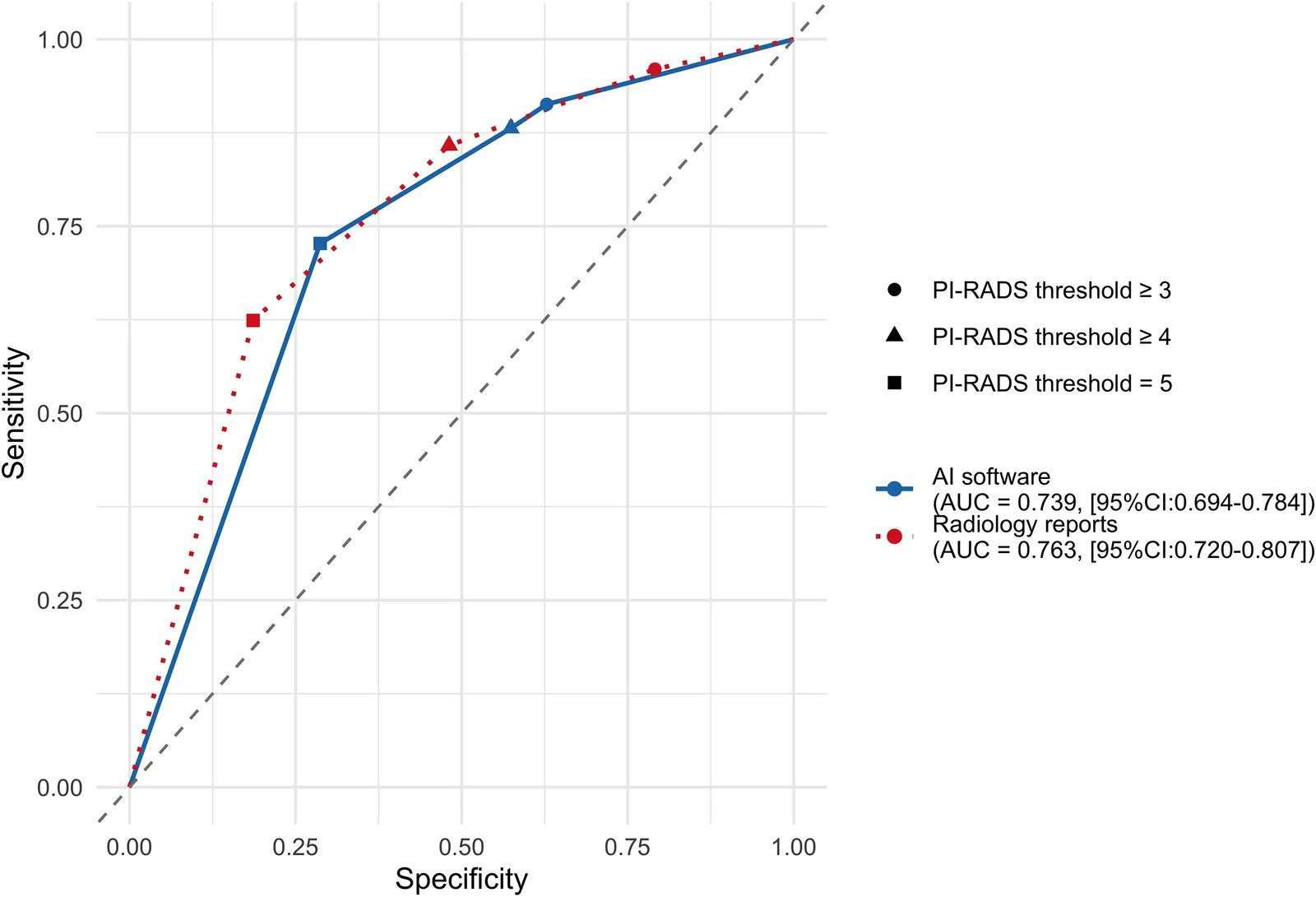
ROC curves comparing the diagnostic performance of the radiology and the AI software readings, with corresponding AUC values.

*Note.* AUC: area under the curve; ROC: receiver operating characteristic.

ROC analysis demonstrated an AUC of 0.763 (range = 0.720–0.807) for radiologists and 0.739 (range = 0.694–0.784) for AI in detecting csPCa. The difference between AUCs was not statistically significant (*P* = .28), indicating comparable overall discriminative performance (Figure 3). Among false-negative cases, 12/33 (36.4%) lesions missed by radiologists measured <6 mm. Among false-positive cases, the majority of lesions identified by both radiologists and AI (91.2% and 90.1%) corresponded to indolent cancers (ISUP grade 1).

In the subgroup of 391 patients who underwent radical prostatectomy, radiologists misclassified 16 (4.1%) examinations as negative, whereas AI misclassified 33 (8.4%). Of these examinations, 10 were concurrently misclassified as negative by both the radiologists and the AI. All false-negative patients were confirmed to harbor csPCa on systematic biopsy and subsequently underwent radical prostatectomy after multidisciplinary review. Paired McNemar testing yielded an odds ratio of 3.83 (95% CI = 1.52–11.51), indicating that AI was nearly four times more likely than radiologists to incorrectly classify an examination as negative. Within this subgroup, only 19 patients underwent multiparametric MRI (mpMRI), and all were classified as positive (PI-RADS 3–5) by the AI software, indicating that differential access to DCE imaging did not contribute to AI false-negative classifications in the McNemar analysis. In a subgroup analysis according to MRI magnetic field strength, the AI software achieved an AUC of 0.783 (range = 0.689–0.877) for 1.5-T MRI and 0.726 (range = 0.676–0.777) for 3-T MRI. The difference in AUC between field strengths was not statistically significant (DeLong's test, *P* = .302).

Finally, a post hoc precision analysis showed that, across the evaluated PI-RADS thresholds, CI widths for sensitivity, PPV, accuracy, and AUC were in the range of 0.027–0.090, whereas those for specificity and NPV were generally wider (Table 3 and Figure 3).

## Discussion

Our results demonstrated comparable overall AUC values between radiology report readings and the tested AI software for detecting csPCa on MRI. However, significant differences in sensitivity and specificity were observed across PI-RADS thresholds, indicating that AUC alone may not fully capture a model's true diagnostic performance.^22^ The relatively narrow CIs for sensitivity, PPV, accuracy, and AUC observed for both radiologists and the AI software in our high-prevalence cohort indicate that these performance estimates were obtained with generally good precision, whereas the generally wider CIs for specificity and NPV indicate greater statistical uncertainty and lower precision for these measures. The AUC values in our cohort for radiologists (0.763) and AI (0.739) were lower than those reported by Saha et al. (0.86 and 0.91), although direct comparison is constrained by methodological and cohort differences.^23^ Nevertheless, our findings are within the ranges reported in previous studies.^12,24^

Both radiologists and AI demonstrated high sensitivity (96% and 91.3%), consistent with previously reported sensitivities in the range of 88%–96.1%.^4,8,23^ In contrast, NPV was lower (0.45 for radiologists; 0.40 for AI at PI-RADS ≥3) than values reported in prior meta-analyses and systematic reviews, where NPV was in the range of 0.61–1.00.^12,25^ This discrepancy likely reflects the high prevalence of csPCa in our cohort (86.5%), whereas earlier studies typically included populations with a lower prevalence, approximately 20%–45%.^15,23^ Further, among the 33 false-negative lesions missed by radiologists, 12 were <6 mm, consistent with evidence that small tumors are more likely to be overlooked on MRI.^26^ Eight were obscured by prostatitis-related signal changes, six by benign prostatic hyperplasia, four by scarring, two were MRI-invisible (both in glands ≤20 mL), and one had reduced conspicuity due to its apical location. Although prior studies have suggested that up to 21% of csPCa may be non-visible on MRI, our false-negative rate was well below this estimate (3.4%).^4,27^ The observed NPVs for both radiologists and AI underscore the importance of considering biopsy or active surveillance when clinical suspicion for PCa persists despite a negative MRI.^5^

The inter-rater agreement between radiologists and AI, as indicated by a Cohen's κ of 0.388, demonstrated only fair concordance. This may partly reflect the higher prevalence of PI-RADS 4–5 lesions compared with categories 1–3, which can influence κ statistics. It also highlights the inherent subjectivity of PI-RADS interpretation, which is limited by modest specificity and significant inter-reader variability.^8–10,28^ Radiologists employed a more conservative approach, assigning more indeterminate PI-RADS 3 scores, while AI generated fewer indeterminate results and a more polarized classification pattern. This observation is consistent with earlier studies where AI models avoided ambiguous categories.^29^ The revised PI-RADS v2.1 system has also been reported to potentially increase the number of indeterminate lesions, which may lead to a higher rate of false-positive findings.^6^ The conservative strategy adopted by radiologists resulted in lower specificity; however, approximately 90% of false-positives corresponded to ISUP grade 1 cancers. Although these are considered clinically insignificant, they still demonstrate histopathologic evidence of malignancy and cannot be entirely dismissed as benign.^30^

The AI software was unable to process 14 MRI examinations due to segmentation errors, such as confusion with adjacent structures (rectum or bowel) or the absence of PI-RADS classification. These limitations emphasize the continued necessity for radiologist oversight, reinforcing AI's function as an assistive rather than autonomous tool.^13^ However, this assistive role is also associated with additional costs. In the subgroup analysis, no statistically significant difference in AI diagnostic performance was observed between 1.5-T and 3-T MRI examinations, despite a numerically higher AUC for 1.5-T MRI. However, the smaller number of 1.5-T examinations should be considered when interpreting these subgroup findings.

To our knowledge, only Bayerl et al. have evaluated the same AI software. Using a threshold of PI-RADS ≥2, they reported 100% sensitivity and NPV for both csPCa and indolent PCa, suggesting the potential to safely avoid biopsies. In contrast, in our cohort, the tested AI software missed one indolent and 12 csPCa cases at the same threshold. This discrepancy likely reflects differences in study populations, as their cohort was smaller (123 vs. 959 patients) and had a substantially lower csPCa prevalence (44.7% vs. 86.5%).^15^

AI has also been proposed to reduce reporting time and triage patients requiring further diagnostic evaluation.^13,31^ However, analysis of the prostatectomy subgroup yielded an odds ratio of 3.83, indicating that the tested AI software was nearly four times more likely than radiologists to classify examinations from patients with csPCa as negative, raising concerns about potential delays in diagnosis and treatment. Taken together, these results underscore that the clinical use of the tested AI software must account for the potential to miss csPCa, particularly in high-prevalence referral populations, which may diminish the software's reliability and argue against AI-driven biopsy selection. Therefore, the software should likely be used only in conjunction with radiologists, thereby limiting potential time savings.

Overall, our findings indicate that although the tested AI software has the potential to support and standardize prostate MRI interpretation, its current limitations necessitate cautious implementation under close clinical supervision.

The present study has some limitations. First, inclusion was limited to patients with histopathologically confirmed PCa on biopsy, which may have resulted in an inflated prevalence of cancer within the study cohort. The study was conducted in a referral center, where disease prevalence is higher than in screening populations, potentially limiting the generalizability of our findings. Radiologists had access to DCE sequences in a subset of patients, whereas the AI software did not use contrast-enhanced series. Among the 124 patients who underwent mpMRI, seven examinations were classified as positive (PI-RADS 3–5) by radiologists but negative (PI-RADS 1–2) by the AI. According to PI-RADS v2.1, however, the formal role of DCE is relatively limited and primarily applies to upgrading equivocal peripheral-zone lesions from PI-RADS 3 to PI-RADS 4. In our dataset, only 9/959 (0.9%) patients represented peripheral-zone PI-RADS 4 assessments in which DCE may formally have contributed to the radiologist score. Notably, the AI software still classified seven of these nine examinations as PI-RADS 4 or PI-RADS 5, suggesting that the impact of differential sequence availability was likely limited. Nevertheless, although this difference was small, it should be acknowledged as a potential contributor to the observed discrepancies. In addition, a small subset of high-risk patients underwent MRI without DCE imaging (*n* = 36). Importantly, none of these examinations were assigned a PI-RADS 3 score; therefore, the absence of DCE imaging would not have altered lesion categorization according to PI-RADS criteria. Although two patients with initially negative MRI findings were subsequently found to harbor csPCa on biopsy, the small number of such cases relative to the overall cohort suggests that the absence of DCE imaging was unlikely to have materially affected the overall study findings. In addition, the analysis was conducted at the patient level without stratification by zonal location. Consequently, a positive AI finding and a positive biopsy result within the same patient could be considered concordant even if they originated from different anatomical regions of the prostate, potentially overestimating the apparent concordance between AI findings and histopathological results. Finally, histopathological results were matched to lesions identified by radiologists rather than those detected by the AI. In future prospective research, hybrid radiologist-AI workflows should be evaluated, and their cost-effectiveness assessed relative to independent readings, to more precisely define the role of AI in clinical practice. Due to the retrospective design of the present study, this aspect could not be investigated.

In conclusion, the evaluated AI software demonstrated diagnostic performance comparable to that of radiologists’ routine readings for detecting csPCa, with no significant difference in AUC. Although AI assigned fewer indeterminate PI-RADS category 3 scores, it also missed more cancers, highlighting important trade-offs that must be considered when integrating AI into clinical practice.

## Supplemental Material

sj-pdf-1-acr-10.1177_02841851261470455 - Supplemental material for Artificial intelligence in prostate MRI: Comparative diagnostic performance in a high-prevalence cohort Supplemental material, sj-pdf-1-acr-10.1177_02841851261470455 for Artificial intelligence in prostate MRI: Comparative diagnostic performance in a high-prevalence cohort by Nádia Gonçalves Ferreira, Owen Matthew Truscott Thomas, Kjell-Inge Gjesdal, Stig Müller, Jan Oldenburg, Ingrid Framås Syversen, Anders Christian Hansen and Jonn Terje Geitung in Acta Radiologica

sj-docx-2-acr-10.1177_02841851261470455 - Supplemental material for Artificial intelligence in prostate MRI: Comparative diagnostic performance in a high-prevalence cohort Supplemental material, sj-docx-2-acr-10.1177_02841851261470455 for Artificial intelligence in prostate MRI: Comparative diagnostic performance in a high-prevalence cohort by Nádia Gonçalves Ferreira, Owen Matthew Truscott Thomas, Kjell-Inge Gjesdal, Stig Müller, Jan Oldenburg, Ingrid Framås Syversen, Anders Christian Hansen and Jonn Terje Geitung in Acta Radiologica
