# Supplementary material for: Artificial intelligence in prostate MRI: Comparative diagnostic performance in a high-prevalence cohort
Source: Acta Radiol. 2026 Jul 27;67(9):765–74. doi: 10.1177/02841851261470455 (PMC13530345; doi:10.1177/02841851261470455)
Supplement: Supplementary file 1 [file sj-pdf-1-acr-10.1177_02841851261470455.pdf]

Prostate Report

mdprostate v.1.3.0

PATIENT ID	NAME	SEX	DATE OF BIRTH	STUDY DATE	SEQUENCE(S)

Prostate Volume and PSA Density

PROSTATE VOLUME [ml]	PSA VALUE [ng/ml]	PSA DENSITY [ng/ml²]
84.50	–	–

Lesion Details and PI-RADS Scoring

LESION	VOLUME [ml]	DIMENSIONS [mm]	ZONE	ADC (MIN/MEAN) [µm²/s]	EPE	PI-RADS OVERALL
#1 (I)	1.1	15.5 × 9.0 × 14.0	PZ	340 / 953	NO	5
#2	< 0.1	4.2 × 2.3 × 2.0	TZ	626 / 767	NO	2

(I) Index lesion

Visual Representation of Slices With the Largest Areas of the 2 Most Important Lesions

Lesion #1

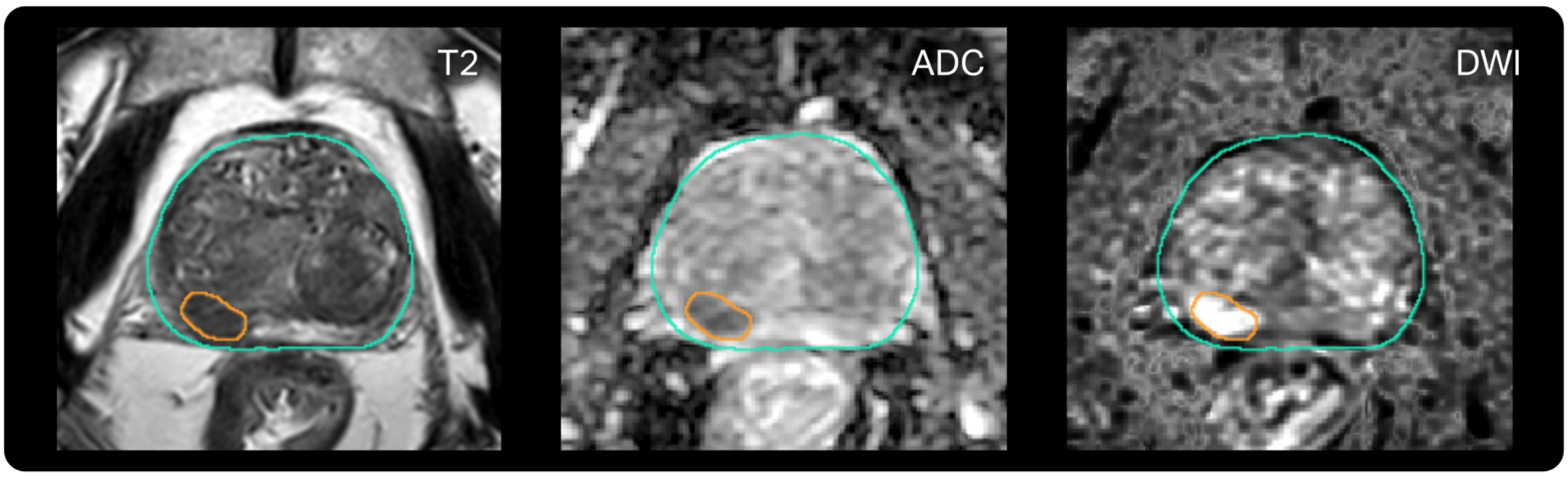

Lesion #1
Lesion #2

Prostate

Lesion #2

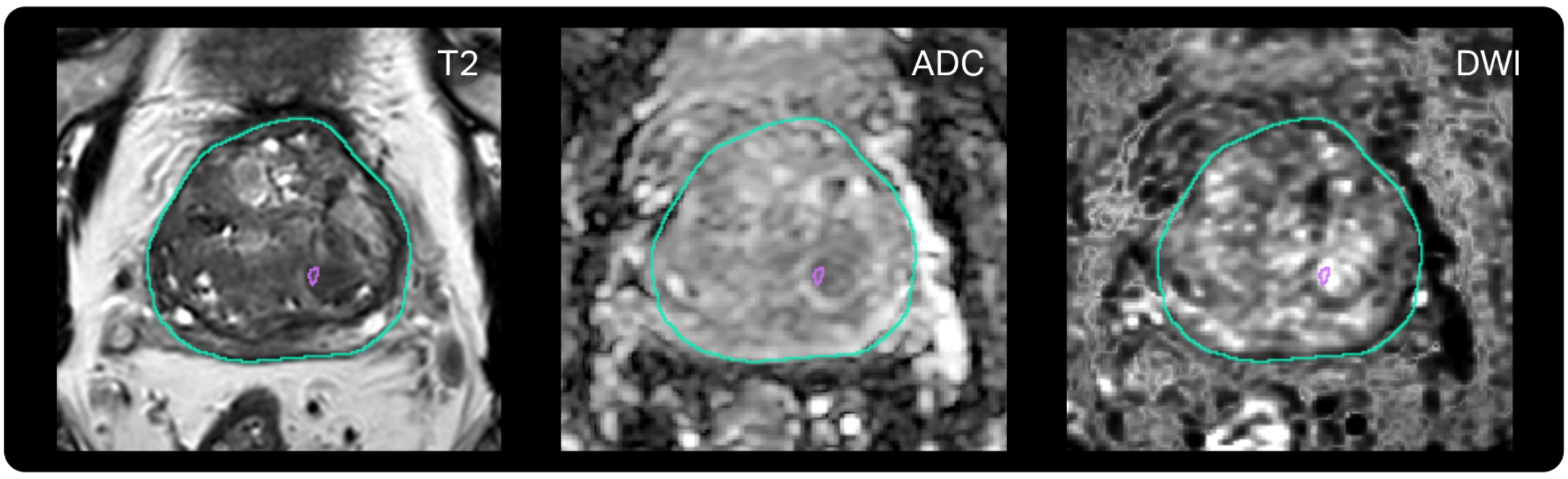

The version of mdprostate you are using is a test version, which is not a medical device and may therefore only be used for test purposes. To install the version released as a medical device, please contact support@mediaire.de.

mediaire
mediaire GmbH info@mediaire.de
Möckernstraße 63, 10965 Berlin

Prostate Report

mdprostate v.1.3.0

PATIENT ID	NAME	SEX	DATE OF BIRTH	STUDY DATE	SEQUENCE(S)

Schematic Mapping of Lesions

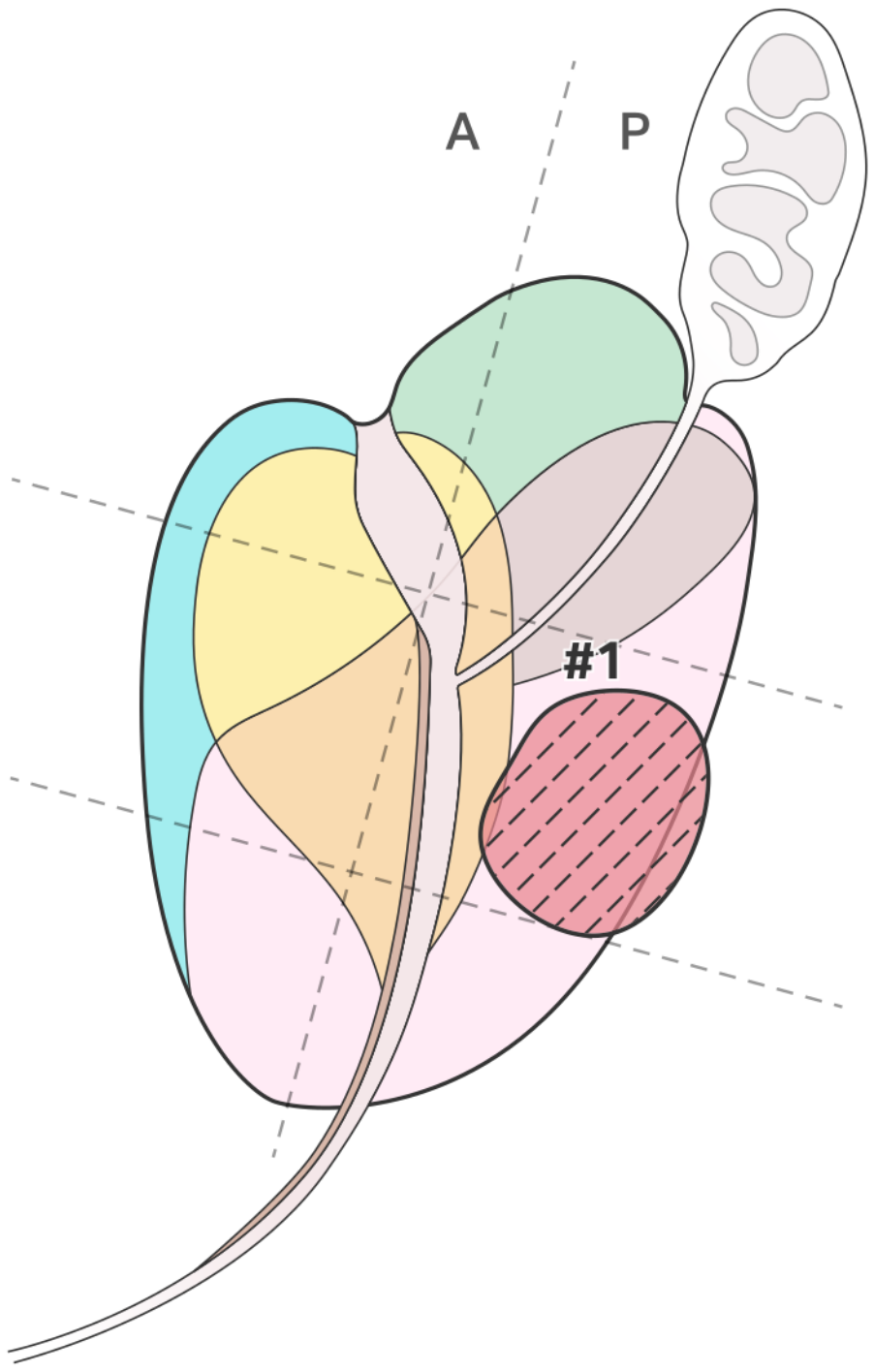

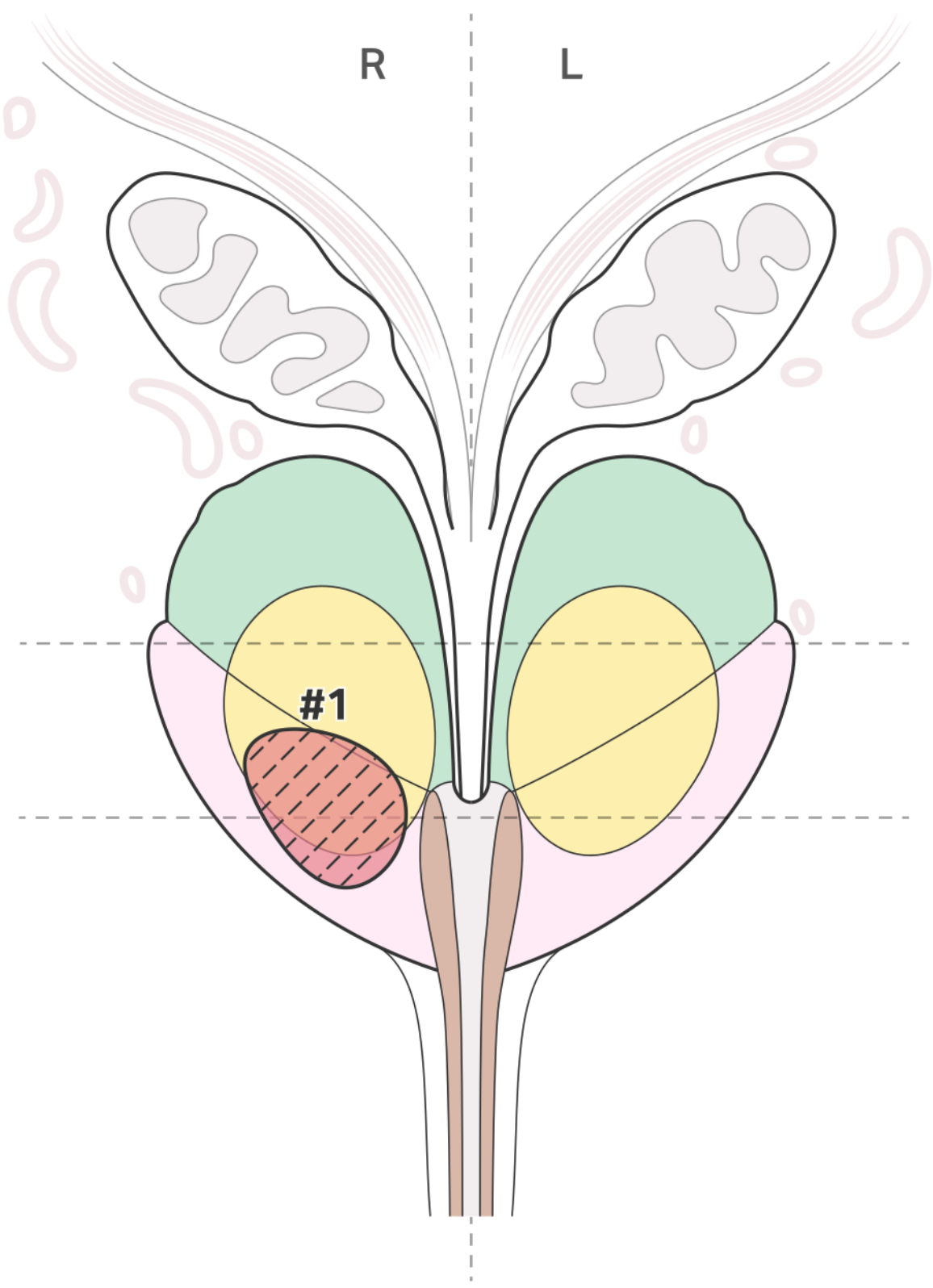

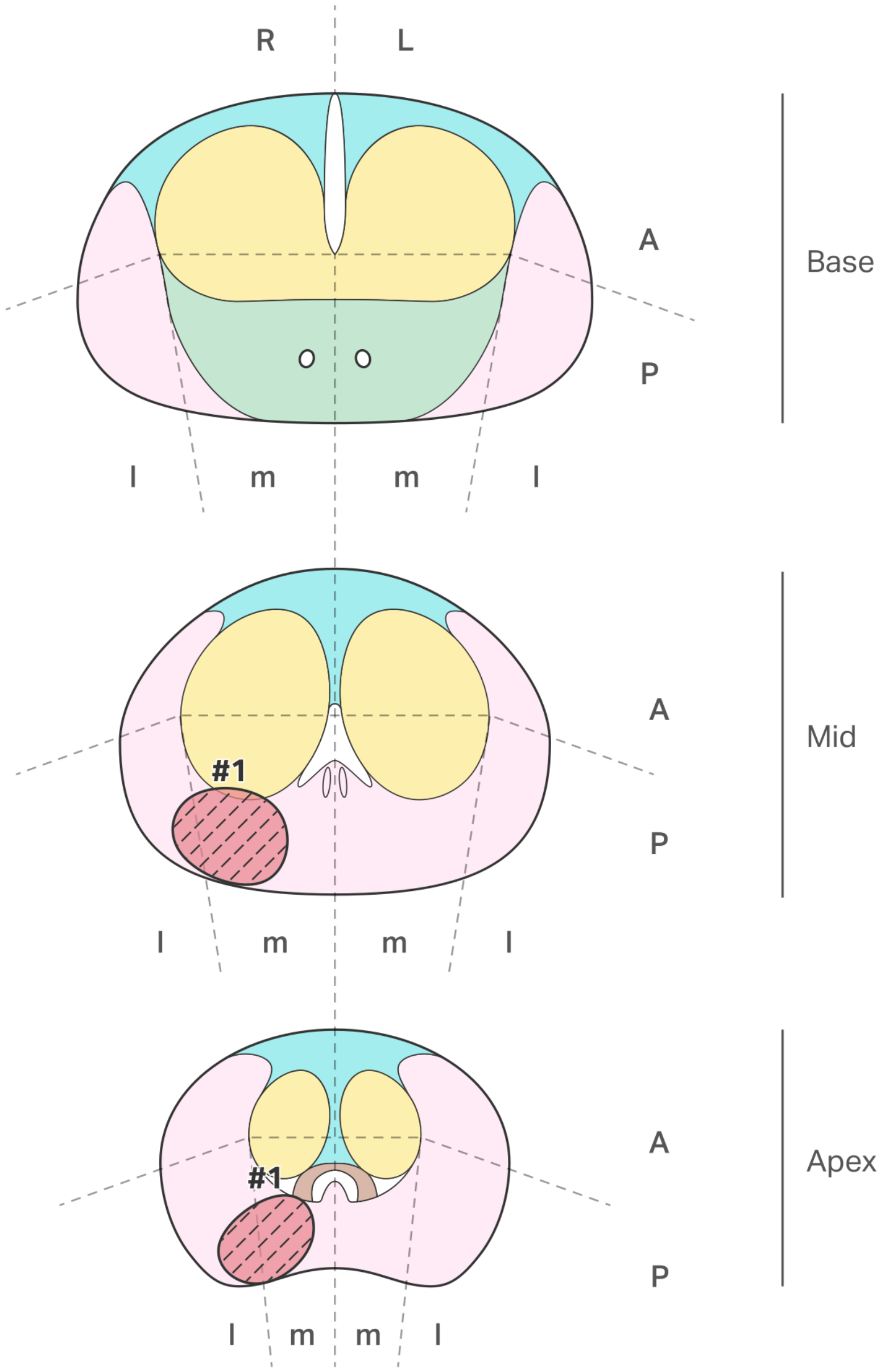

- PI-RADS score 5
- PI-RADS score 4
- PI-RADS score 3

- PZ
- CZ
- TZ
- US
- AFS

Quality Control

IMAGE QUALITY	Very good
NOTES	None

The version of mdprostate you are using is a test version, which is not a medical device and may therefore only be used for test purposes. To install the version released as a medical device, please contact support@mediaire.de.

mediaire
mediaire GmbH info@mediaire.de
Möckernstraße 63, 10965 Berlin
