# Supplementary material for: Artificial intelligence in prostate MRI: Comparative diagnostic performance in a high-prevalence cohort
Source: Acta Radiol. 2026 Jul 27;67(9):765–74. doi: 10.1177/02841851261470455 (PMC13530345; doi:10.1177/02841851261470455)
Supplement: Supplementary file 2 [file sj-docx-2-acr-10.1177_02841851261470455.docx]

Table S1. Overview of sequence parameters for the 3 image weightings used by the AI software - T2-weighted, diffusion-weighted image with b = 1500 s/mm², and ADC map. Values in the table are given in min-max range.

|  | 1.5 T (n = 176 patients) | | | 3.0 T (n = 783 patiens) | | |
| --- | --- | --- | --- | --- | --- | --- |
|  | T2w | DWI b1500 | ADC | T2w | DWI b1500 | ADC |
| TR (ms) | 1200 - 4574 | 2588 –  8479 | 2398 –  8340 | 1200 - 4986 | 2018 –  8577 | 2476 - 8577 |
| TE (ms) | 110 - 229 | 50 - 110 | 59 - 86 | 120 - 213 | 47 - 90 | 53 - 90 |
| In-plane resolution (mm^2^) | 0.3 - 0.5 | 0.5 - 2.7 | 0.5 - 1.3 | 0.3 - 0.5 | 0.5 - 1.3 | 0.5 - 1.3 |
| Slice thickness (mm) | 0.9 - 3.5 | 3.0 - 4.0 | 3.0 - 4.0 | 0.9 - 3.5 | 3.0 - 5.0 | 3.0 - 5.0 |

- 7 MRI machines used:
  - Philips Achieva dStream 1.5 T
  - Philips Achieva dStream 3.0 T
  - Philips Ingenia 1.5 T (n = 3 machines)
  - Philips Ingenia Ambition X 1.5 T
  - Philips Ingenia Elition X 3.0 T
- For T2-weighted imaging:
  - 522 3D acquisitions
  - 437 2D acquisitions
- For DWI b1500:
  - 385 patiens had a calculated b-value of 1500 s/mm² based on a standard DWI acquisition with 2 b-values
  - 574 patients had a separate diffusion acquisition with a single b-value of 1500 s/mm²
- ADC (apparent diffusion coefficient) map calculated from a diffusion sequence with 2 b-values:
  - Minimum b-value in range 0-50 s/mm²
  - Maximum b-value in range 800-1000 s/mm²
